# Impact of Changes in Criminal-Legal Practices During the COVID-19 Pandemic on the HIV Risk Behaviors of Women Who Use Drugs: Protocol for a Multimethods Qualitative Study

**DOI:** 10.2196/58285

**Published:** 2024-12-20

**Authors:** Amy B Smoyer

**Affiliations:** 1 Department of Social Work Southern Connecticut State University New Haven, CT United States

**Keywords:** HIV, corrections, women, substance use, COVID-19, criminal-legal systems, carceral, qualitative, SARS-Cov-2, coronavirus, pandemic, drug, women who use drugs, psychosocial, HIV risk, health care, qualitative method, socio-demographic, audio-recorded, thematic analysis

## Abstract

**Background:**

HIV risk behavior in women who use drugs is related to myriad psychosocial issues, including incarceration. The experience of incarceration elevates women’s HIV risk by disrupting social networks, housing, employment, and access to health care. During the COVID-19 pandemic, changes in criminal-legal practices resulted in decreased incarceration, especially among women. These changes may have largely altered HIV risk among women who use drugs, depending on their access to care in the community.

**Objective:**

This study seeks to build knowledge about the impact of shifts in criminal-legal practices during the COVID-19 pandemic on HIV risk behaviors of justice-involved women who use drugs.

**Methods:**

Qualitative methods are used to gather and analyze women’s narratives about their life experiences before and during the COVID-19 pandemic, with a focus on individual and structural determinants of HIV risk behaviors. Thirty formerly incarcerated women with a history of substance use are being recruited through collaboration with community partners. Each participant completes a sociodemographic survey and two interviews. The first interview uses a life history instrument that invites participants to reflect on key turning points in their lives. The second interview uses a calendar approach to gather information about participants’ lives during the first year of the COVID-19 pandemic (March 2020-March 2021). The interviews (1 hour each) are audio-recorded and transcribed for analysis. Rapid Qualitative Inquiry and thematic analysis are being used to manage, organize, and interpret the data. The study team will collaborate with a subset of participants to develop digital stories about their COVID-19 experiences, a process that allows for member-checking and triangulation. Findings will be disseminated to program and policy makers in academic venues, community settings, and social service agencies.

**Results:**

To date, 10 women’s data have been collected. In total, two themes have been identified in this preliminary data: (1)the chaos and instability of participants’ lives increased during the COVID-19 pandemic: participants reported a wide range of psychosocial and health problems and limited engagement with social service systems. Interaction with criminal-legal systems was rife with uncertainty; participants described living in a state of limbo, which was extremely stressful. (2) When asked to describe a “turning point” in their lives, many participants attributed their substance use to the traumatic loss of a child due to death, incarceration, or termination of parental rights. During the COVID-19 pandemic, participants’ struggles to cope with these unresolved experiences of grief and loss were intensified by the widespread death and dying of the pandemic.

**Conclusions:**

Preliminary findings suggest that HIV risk factors increased for participants during the COVID-19 pandemic and invite further investment in community-based harm reduction programs, especially housing, that support women who use drugs. Interventions that address experiences of maternal grief and loss may reduce women’s substance use.

**International Registered Report Identifier (IRRID):**

DERR1-10.2196/58285

## Introduction

### Background

In 2019, a total of 6400 women were diagnosed with HIV in the United States, representing 18% of the country’s new infections [1]. About half of these new infections were among African American women [2]. Women who use illicit drugs are also disproportionately impacted by HIV: 16% of newly diagnosed women in 2019 attributed the transmission to injection drug use and women who use drugs are more likely to report high-risk sexual behavior [2-6].

The ability of women who use drugs to reduce their HIV risk (ie, negotiate condoms, reduce their number of sexual partners, use sterile syringes, and access pre-exposure prophylaxis) is mediated by a constellation of factors related to the individual and structural determinants of human behavior [6]. This project focuses on one of these myriad factors, incarceration, because women who use drugs, especially women of color who use drugs, are disproportionately ensnared in carceral systems. There are approximately 170,000 women incarcerated in the United States, a number that has increased 7-fold since 1980 [7]. Compared with white women, the rate of incarceration is 1.7 times higher for Black women and 1.3 times higher for Latinas [7]. Most incarcerated women meet the criteria for a substance use disorder, and report using drugs in the 30 days before their arrest [8,9]. Women are primarily convicted of nonviolent drug-related, property, or public order crimes [10].

Research has documented how incarceration elevates HIV risk by disrupting housing, employment, relationships, and access to health care, including substance use treatment programs [11-18]. These findings recommend services that can be provided during and after incarceration to recover from carceral disruptions and trauma [19-23]. However, resources are limited for the thousands of women who are released each year [24]. Furthermore, consensus around the detrimental impacts of incarceration on HIV risk has failed to articulate if and how community-based services could address individual and community harm, including HIV risk, in the absence of criminal-legal sanctions.

Research has documented the perception among criminal-legal professionals, service providers, and even women who use drugs that prison is the only place that can provide women in crisis with safe refuge [25,26]. Indeed, incarceration may temporarily decrease harm, including HIV risk, for women who use drugs by interrupting drug use, unprotected sexual behavior, interpersonal violence, homelessness, and hunger. That some women describe prison to be “the safest place I have ever lived,” despite the abundant research that documents the trauma and stress of incarceration, speaks to the profound danger and vulnerability experienced by marginalized women who use drugs in the United States [25]. Efforts to decrease the use of incarceration as a response to illicit behavior among women who use drugs must develop community-based alternatives to incarceration that can protect and support women who are actively using or experiencing serious mental health episodes [27].

Currently, the term “alternatives to incarceration” is used to describe community-based criminal-legal responses, that is, probation, mandated treatment, drug court. The largest alternative to incarceration is probation, a legal mechanism that sentences people convicted of crime to community supervision, allowing them to stay in the community provided they adhere to the stipulations of their supervision. There are about 700,000 women on probation in the United States [28]. An assessment of this population’s needs and HIV risk has found that “compared to those with no CJ [criminal justice] involvement…women who had been on probation or parole in the past year had higher adjusted odds of homelessness, physical assault, sexual assault, illegal income and injection drug use in the past 6 months” [29]. These findings suggest that probation is not effective in reducing women’s HIV risk behavior. Furthermore, probation often leads to incarceration: 29% of prison admissions in 2019 were for violation of community supervision rules [30], further elevating this group’s HIV risk, as previously noted [11-18].

### COVID-19 Pandemic: a Natural Experiment in Criminal-Legal Reform

Short-term changes in criminal-legal policies during the COVID-19 pandemic created a natural experiment about the impact of criminal-legal, social service, and community-based alternatives to incarceration on the HIV risk of women who use drugs. In 2020, many states sought to prevent the transmission of COVID-19 by decreasing the number of people detained in carceral institutions [30-32]. Connecticut led the nation in this decarceration response, reducing its prison population by 26%, the largest drop of any state [32]. The number of people at Connecticut’s correctional institution for women decreased by 43% between March 01, 2020, and March 01, 2021, from 883 to 502 women [33]. While some women were released early, this decrease in prison population was primarily related to changes in policing, prosecution, and judicial procedures that slowed entry into the state’s correctional facilities [31,32]; more specifically, there was a large decrease in the number of women held in pretrial detention and serving sentences of less than a year for minor nonviolent charges (eg, violation of probation, drug sales or possession, sex work, larceny) [33]. Drug-related behaviors that would have resulted in incarceration before the COVID-19 pandemic were largely overlooked during this time.

The impact of these changes in criminal-legal practices during the COVID-19 pandemic on the HIV risk behaviors of women who use drugs is not known. Nascent research suggests that access to community-based programs varied, creating disparate experiences. For example, to promote social distancing, methadone clinics implemented systems of “take home” doses for all clients and eliminated the requirement for psychosocial groups [34]. Lifting rules which required clients to come in person each day to receive methadone and participate in groups may have facilitated access to the medication for some, while jeopardizing the recovery of those who relied on program structure and group support. Similarly, housing stability increased for some unhoused people who were offered long-term stays in local hotels to reduce loitering and sleeping in public areas and to allow people with COVID-19 infection to quarantine [35,36]. However, these isolated accommodations may have had some detrimental outcomes, for example, reduced sense of community belonging and social connection [35-38]. Overdose trends suggest that the COVID-19 era was dangerous for people who use drugs, especially non-Hispanic Blacks. The number of fatal overdoses in Connecticut increased by 14.3% during 2020 [39]. Qualitative data from women who use drugs about their lived experiences during the COVID-19 pandemic can help build knowledge about what happened during the pandemic and how the shifting service environment shaped their HIV risk.

## Methods

### Overview

This community-engaged research project uses multiple qualitative methods to explore the lived experience of justice-involved women who use drugs (n=30) to answer the research question: what was the impact of COVID-19–related shifts in criminal-legal practices in CT on the HIV risk behaviors of justice-involved women who use drugs? Study methods include life history interviews, calendar interviews, and digital storytelling. The team-based approach to data collection, analysis, and dissemination reflects the project’s commitment to undergraduate mentorship and training and brings an intentionality to each step of the research that slows down the process and allows for deep contemplation of the data.

#### Conceptual Framework

**Figure 1:**
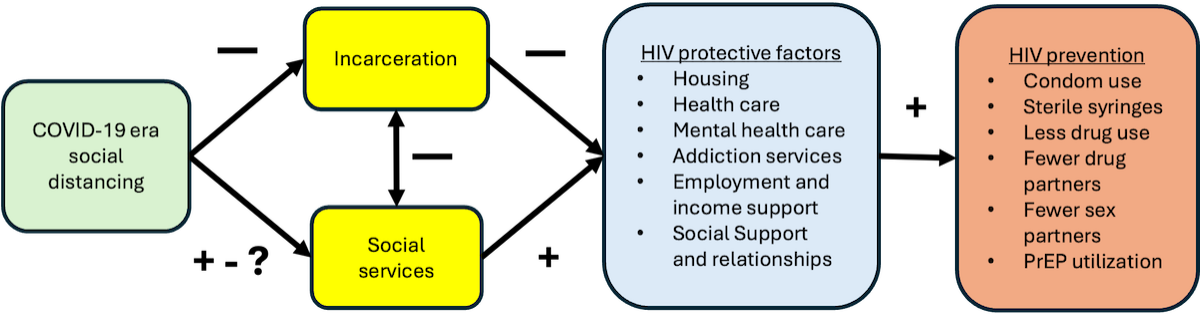
Conceptual framework. PrEP: pre-exposure prophylaxis.

Figure 1 illustrates this project’s conceptual framework. During the COVID-19 pandemic, social distancing mandates decreased the use of incarceration in Connecticut to avoid crowding inside carceral institutions. These mandates also impacted community-based social services. Some community programs expanded to provide emergency support, while others contracted to protect the health of clients and staff [40]. Given that incarceration is negatively associated with HIV protective factors, the limited detention of women who use drugs during COVID-19 pandemic may have reduced their HIV risk. However, for women who use drugs in the community who are experiencing acute addiction, mental health, and socioeconomic crises, HIV risk would only be reduced if they were willing and able to access psychosocial services, housing, health care, and other needed supports [41]. The dramatic decrease in the incarceration of women in Connecticut during COVID-19 pandemic offers an opportunity to explore this conceptual framework by building knowledge about what happened to justice-involved women who use drugs when they were not incarcerated.

#### Study Funding and Team

This study is funded by an R15 grant from the National Institutes of Health’s National Institute on Drug Abuse (NIH/NIDA 1R15DA056285; Principal Investigator [PI]: ABS) (Multimedia Appendix 1). The R15 mechanism has a dual mission to build health knowledge and create research opportunities for undergraduates studying at institutions that have not been major recipients of NIH funding [42]. From this, the study team, which began meeting in June 2022, is comprised primarily of undergraduate students. Led by the PI, who is a member of the university’s social work faculty, the team consists of 5 to 7 student research assistants (RAs) and a part-time graduate assistant. In addition, three community practitioners, who are alumnae of the university’s social work program and have expertise related to substance use, criminal-legal systems, trauma, and HIV, serve as project consultants. The team is primarily cisgender women and nonbinary people and includes women with lived experiences of substance use and incarceration. Over half of the study team are Black and Latinx.

### Participant Recruitment

Participant recruitment began by engaging with women who use drugs and the service providers who support this population. Since the beginning of the project, the research team has been visiting with local service providers, volunteering with community-based agencies, and attending local re-entry roundtable and HIV planning meetings. These experiences have allowed research staff to spend time with justice-involved and formerly incarcerated women who use drugs, become familiar with local organizations that support justice-involved women, and notice and reflect upon their own positionality. In June 2023, after a year of this community engagement, the team began to recruit participants using stratified convenience sampling. The goal is to recruit up to 30 people who (1) have been incarcerated at the York Correctional Institution (CI) in Connecticut at least 3 times before March 2020, and (2) report a history of drug use (ie, have received in-patient or out-patient drug treatment, experienced overdose, or were incarcerated or placed on probation for a drug-related crime). The exact number of people who enroll in the study will depend upon when saturation is achieved (ie, interviews do not introduce new information or themes to the project) [43]. Research has found that saturation is generally reached with 12-17 individual qualitative interviews [43,44]. The diversity of this population and their lived experiences suggests the sample size at which this project reaches saturation may be slightly higher than average [44].

York CI is the only carceral facility designated for women in the State of Connecticut. It is a consolidated facility that serves both jail and prison functions, incarcerating people for pretrial detention and short- and long-term sentences. While most of the people detained in this facility are cisgender women, the population may also include nonbinary people, transgender men, and transgender women. This study has no eligibility criteria related to gender identity: anyone with a history of drug use who was incarcerated at York CI at least 3 times before the COVID-19 pandemic is eligible to participate. The sample will be stratified by race, oversampling Black and Latina women, who are disproportionately impacted by HIV, to construct a sample that is 50% Black, 30% Latina, and 20% White. This recruitment plan has been developed and implemented in collaboration with community consultants.

Recruitment flyers have been distributed to local service agencies that work with formerly incarcerated people in New Haven, Connecticut. Potential participants can express interest by contacting the study team by telephone or by scanning the QR code on the recruitment flyer to complete an online interest form. Snowball sampling, wherein participants share information about the study with their friends and colleagues, has also been encouraged. Study team members use a screening tool to determine the eligibility of all potential participants who contact the study. This 11-item survey can be administered by phone or in person. All eligibility information about individual histories of incarceration and drug use is based on self-report data. Contact information is collected from eligible participants. This information includes first names, telephone numbers, and email addresses (if any), and the option to provide the name of a professional support person who the study team could contact if phone and email are not working. This contact information is recorded in a password-protected Excel (Microsoft Corp) spreadsheet that is not linked in any way to the study data and is used only to communicate with participants about scheduling data collection appointments.

### Data Collection

After establishing a participants’ eligibility, staff explain that the project requires three consecutive appointments as well as the option to participate in a digital storytelling project. If an individual wants to participate, a first appointment is set, usually for the following day. At this first appointment, informed consent is administered. During this process, staff emphasize that participation is voluntary, and participants can withdraw from the study at any time and decline to answer any question. The consent process and all data collection are conducted in a private office at a centrally located social service agency that provides housing, employment support, and case management to justice-involved people. This location is well known by the community and easy to access by public transportation.

All data collection is conducted by two team members, using “joint interviewing” [45]. Each RA will participate in four interviews. During the first two of these interviews, the PI conducts the interviews with the undergraduate RA serving as an observer and note-taker. During the next two interviews, the RA will conduct the interviews with the PI serving as an observer and note-taker. This graduated system of support builds RAs’ research capacity by offering mentorship, feedback, and practice. It also strengthens the project to have more than one person conducting the interviews, allowing multiple perspectives and experiences to shape the data.

The study includes three data collection points: the sociodemographic survey, life-history interview, and COVID-19 calendar interview. These data collection points each take 30-90 minutes to complete and are conducted on separate days. To compensate for their time and expenses, participants are compensated US $20 at the survey meeting and US $40 for each interview. In addition, participants have the option to participate in a digital storytelling project after completing the survey and interviews.

The sociodemographic survey, which was built in Qualtrics (Silver Lake) and includes 141 close-ended questions, is administered using an iPad immediately after consent is administered. Participants can complete the survey on their own or can choose a staff-administered survey option. The survey takes about 10 minutes to complete when self-administered and 20 minutes when staff administered. The survey questions ask about demographics, family, housing, employment, health, criminal-legal experiences, and substance use and treatment.

Individual life history interviews use a semistructured instrument to understand women’s lives before the COVID-19 pandemic [46]. These 90-minute interviews begin by asking participants to identify key “turning points” in their lives [47]. They are then invited to describe these critical periods. Follow-up questions ask participants to discuss the psychosocial contexts (family, education, employment, housing, and social services) of these moments with a focus on criminal-legal, drug use, and sexual histories. Data from these life history interviews establish women’s baseline behaviors before the COVID-19 pandemic. The interview is intentionally strengths-based and designed to build rapport between the participants and study team [48,49].

The calendar interview focuses on participants’ lives during the first year of the COVID-19 pandemic (March 2020-March 2021). Using calendar techniques, specific information is gathered about participants’ socioeconomic circumstances, with a focus on criminal-legal experiences and HIV risk and protective factors [50]. The interview begins by asking participants to review a 20-21 calendar, noting anchoring dates from their own lives and the US calendar that spark memories about this period [51]. Next, study staff ask a series of specific prompts about their lived experience during this time, with a focus on HIV protective factors, sexual behavior, and drug use.

### Digital Storytelling and Member Checking

At the end of the calendar interview, staff ask participants if they want to create a digital story related to their life during the COVID-19 pandemic. Guided by Lambert’s Digital Storytelling Cookbook, the study team will run a series of individual digital storytelling sessions with interested participants [52]. The storytelling process will take 4 to 6 sessions to complete, and participants will be compensated US $20 per session for time and expenses. The PI and one RA, who have both completed StoryCenter’s Digital Storytelling training, will teach the study team, including the community consultants, how to facilitate these sessions [53]. Digital stories are a tool that allows people to share moments in their lives through short, 3- to 4-minute, narrated videos [54]. Participants’ digital stories will provide another data point to triangulate narratives collected in the COVID-19 calendar interviews. The process is also an opportunity for member-checking, an activity that builds the trustworthiness of qualitative data. Finally, with participants’ permission, digital storytelling can be a mechanism for dissemination and community engagement.

The first digital storytelling session will be dedicated to member checking, a process that builds the trustworthiness of qualitative study findings by gathering participant feedback and comments on analyzed study data [55]. Participants will be given a copy of their transcribed interviews and a summary of preliminary findings and quotes that exemplify each finding. Members of the study team will discuss the findings, consider if the data align with their memories, and invite participants to comment on anything they would like to change or add [55]. This member checking has 2 purposes: (1) participants’ feedback will inform and validate the ongoing data analysis process; (2) the process will refresh participants’ memories about the COVID-19 era in preparation for the digital storytelling process. In subsequent sessions, the team will guide participants to write a short story and produce a short video using still photos. Participants may choose to build upon a narrative in the calendar interviews or pick another moment in time. Digital storytelling tasks will include imagining, writing, planning, video instruction and editing, sharing, and reflecting [52,54].

### Data Analysis

Data analysis will be conducted in two rounds. First, data collection will pause after every block of 5 interviews to conduct Rapid Qualitative Inquiry (RQI). Second, after all data have been collected and transcribed, thematic analysis of the interview data will be conducted.

#### Rapid Qualitative Inquiry

RQI is a method of data collection, management, and analysis that centers the knowledge of impacted community [56]. RQI is defined as: “intensive, team-based qualitative inquiry with (a) a focus on the insider’s or emic perspective, (b) using multiple sources and triangulation, and (c) using iterative data analysis and additional data collection to quickly develop a preliminary understanding of a situation” [57]. RQI processes concentrate the data in a way that allows for a rapid analysis without compromising rigor or trustworthiness. This express treatment of the study data will allow for prompt feedback for social service and state agencies about the impact of the COVID-19 era on women’s HIV risk in order to inform prevention services. Given the rapid evolving nature of the COVID-19 pandemic, and the possibility of other viral outbreaks, this technique offers a way to give immediate knowledge and information to relevant agencies. This approach will also allow the student RAs to practice using a data analysis tool that aligns with social work’s need for swift, pragmatic, and accurate understandings of program outcomes.

As part of the RQI process, data are collected in “scheduled blocks,” a strategy that sets intentional pauses in data collection for the team to review data and consider possible conclusions and what changes, if any, should be made in subsequent interviews [58]. After each set of 5 interviews, the entire team will meet to discuss this set of interviews, for a total of 6 data collection blocks [59]. Presentation of the interview data at team block meetings will begin the analysis process and allow for early identification of central themes and patterns that can inform subsequent interviews. In preparation for these block meetings, the audio from each interview will be transcribed using Rev, an online transcription service. Next, each transcription will be reviewed and edited by the RA who participated in the interview. During this review process, all identifying information, including names and specific locations, will be removed from the text. The RA will then create a summary of the interview that will be shared with the team and presented at the block meeting [56]. The summary will provide a sociodemographic description of the participant and her reported HIV risk behaviors (individual and structural) and criminal-legal experiences. The goal is to create “intensive team interaction” among the study team throughout the data collection and analysis process [60]. A matrix will be developed by the team, based on discussion at the meetings, that summarizes participants’ pre–COVID-19 experiences.

#### Thematic Analysis

Thematic analysis is an iterative process that uses a “family of methods” to summarize and interpret participants’ narratives [61]. The study team will begin its thematic analysis with a series of steps that develops a summary of the topics that are explored and explicated in the data. At the same time, team members will simultaneously engage in a reflective analysis and interpretation of the data that identifies and articulates deeper meanings and ideas within the participants’ narratives.

#### Topic Summary

After the interviews have been conducted and transcribed, the transcripts will be uploaded into Dedoose (SocioCultural Research Consultants), a qualitative data management application for thematic analysis [62]. To begin this analysis, the team members will use the thematic analysis steps set forth by Braun and Clarke [63]. While Braun and Clarke [64] caution against following these steps like a “baking recipe” (ie, a set of procedures to be followed), the topic summaries that the team creates with this analysis will offer a valuable orientation to data analysis for the team’s novice undergraduate RAs. In addition, these summaries will offer a description of women’s lived experiences during the COVID-19 pandemic related to drug use, sexual behavior, housing, food security, and access to health and social services that can begin to inform social work practice. The first step in this process is to become familiar with the data. Because the team conducted the interviews and reviewed and summarized the data during the RQI process, they will already be acquainted with the interview data. The next step is to generate an initial set of codes (or, in this case, topics), based on the project’s research questions, and team notes and summaries about the content of the data. This scheme of initial codes, which are likely to have a positivist slant, will then be piloted in Dedoose, with each team members coding one transcript. After this initial application, the team will meet to review, edit, and further define each category. The team will then return to the dataset to code the remaining interviews; each interview will be independently coded by two people. Once the data have been coded, the team can develop findings by extracting and analyzing data that have been coded at one or more themes.

#### Reflective Analysis

While the team is coding to create topic summaries, they will also be invited to engage in reflective thematic analysis [65]. The PI will be reading the narratives in an interpretive manner with the goal of identifying latent meaning. As a social worker with two decades of experience working with justice-impacted individuals on issues related to HIV prevention, the PI has a personal and professional framework through which she will interpret and derive meaning from the data. This reflective analysis is a subjective practice that will be informed by input from colleagues. Discussion with the full research team, including undergraduate RA and community consultants, will help to curate and refine these interpretations. Student RAs who can reach the “depth of engagement” [66] that is required to conceptualize the data in this nonpositivist manner may also contribute reflective analysis of the data. The level of analysis that is available to these new scholars, who are juggling myriad personal, professional and academic responsibilities, is an empirical question that this collaboration can begin to answer.

### Trustworthiness and Credibility

Several processes are being taken to ensure the trustworthiness and credibility of these qualitative data. One, team members will engage in deliberate reflection to recognize and examine their positionality through open researcher-participant interaction, peer debriefings, and reflexive journaling [67]. Weekly meetings create space for peer dialogue and critical thinking while participation in community forums brings RAs in close contact with women who use drugs and creates opportunity for them to explore their own ideas about and reactions to criminal-legal, addiction, and HIV-related topics. Two, the study’s data collection, analysis, and reporting steps are designed to promote the rigor of study findings. By engaging a diverse team in all aspects of the project from development of study instruments to data collection, the validity of the study is boosted [67]. Sampling techniques and goals are designed to establish broad representation and scope. Oversampling populations most impacted by HIV promotes the usefulness of data. A team approach on data collection and analysis encourages group and individual reflection about research-participant interactions and transparency [56]. The calendar and DST techniques encourage the creation of thick descriptions with rich details as specific moments in time are explicated. During the data analysis process, efforts will be made to remain open to the possibility of conflicting or contrasting narratives that run counter to overall themes [67]. Specific findings that can inform actionable next steps and be applied to other contexts will be advanced.

Efforts will also be made to bolster the credibility of this study [68]. First, the PI and the study consultants have been engaged with communities impacted by criminal-legal systems and HIV for decades. These relationships have established a track record that strengthens the project’s credibility. In addition, the university with which the PI, community consultants, and student RAs are affiliated is a public, regional university of access with strong reputable ties throughout the community. Precise record keeping of the study’s confidential dataset will allow study narratives to be available to other researchers who want to work with or examine the data.

### Ethical Considerations

The protocol was reviewed and deemed exempt by the Southern Connecticut State University institutional review board (protocol #670). Informed consent is administered to all interview participants. During this process, it is emphasized that participants are volunteers and can decline to answer any questions or end the interview at any time. Specific permission to audiotape the interview is requested and each participant is invited to select their own pseudonym. During the transcription process, all identifying information (ie, names, dates, and locations) are removed. To protect participants’ confidentiality, consent is obtained verbally because writing the participants’ names on the consent form would create the only record of their name in the study files.

## Results

### Overview

Ten cisgender women have completed the survey and both interviews. Participants identify as Black (n=5), Latina (n=4), and White (n=1). Their average age is 40 (range 27-56) years. Data collection will be completed by December 2024, with analysis and dissemination continuing through 2025. Analysis from the first “scheduled blocks” have resulted in 2 preliminary findings. These preliminary findings suggest that the circumstances of women who use drugs deteriorated during the COVID-19 pandemic, and that services that might reduce HIV risk behaviors were not accessed.

### Instability

The chaos and instability of participants’ lives increased during the COVID-19 pandemic. Most experienced homelessness and were unable to access COVID-19–related housing programs or were evicted from these programs after short stays. Participants reported that social isolation and pandemic-related fear increased their anxiety, depression, and substance use. Sources of income included minimum-wage employment, primarily at fast food outlets, and illicit hustles, including sex work and larceny. Use of social services was low; food pantries and mobile syringe services were the most frequented programs. Several women were arrested during the COVID-19 pandemic and were either sentenced to probation or released with a “promise to appear” in court at a future time for adjudication. The uncertainty of being placed on probation with unclear stipulations and awaiting future court dates fueled a sense of hopelessness that increased substance use. Women reported that they frequently had limited to no cell phone access, which hindered their ability to maintain communication with judicial and social service systems. These findings suggest that community services failed to protect and support women who use drugs who were experiencing acute mental health and substance use crises during the COVID-19 pandemic.

### Grief and Loss

When asked to describe a “turning point” in their lives, most of the women talked about the traumatic loss of a child. Participants’ experiences of loss included the death of a child to illness or accident, the long-term incarceration of a child, and the permanent, sudden removal of one or more children by the state’s child protection agency. None of the participants had ever received any kind of psychosocial services related to their maternal grief and loss. Left alone to cope with these experiences, many women reported using substances and engaging in self-harm behaviors. While none of the participants reported losing a close family member or friend to COVID-19 infection, bearing witness to the widespread death and dying of the pandemic activated their own unresolved grief and surfaced a range of complicated emotions that they were unprepared to manage. This finding informs knowledge about the etiology of substance use and invites further research and program development related to mothers’ grief related to the loss of a child. In addition, this data is an opportunity to better understand how noncustodial mothers employ narratives of grief and loss to perform and assert their identity as “good mothers” in a socioinstitutional environment that has consistently denied or contested this claim.

## Discussion

### Summary

This project investigates the lived experiences of women who use drugs during the COVID-19 pandemic, when the use of incarceration decreased, and asks: What happens when she is not incarcerated? Given that incarceration is understood as a structural determinant of women’s HIV risk, one hypothesis is that the HIV risk of women who use drugs may have decreased when they remained in the community with their sexual and drug using networks intact. Alternatively, HIV risk may have increased due to social isolation, stress, and reduced access to community-based services, including substance use and mental health treatment. Similarly, postponement of criminal-legal adjudication or punishment may have offered respite from prison life or could have been experienced as a time of uncertainty and limbo that elevated women’s anxiety and risk behavior. This qualitative study uses multiple methods to better understand how COVID-19 era shifts in criminal-legal practices impacted the HIV risk behaviors of justice-involved women who use drugs.

### Preliminary Findings

Preliminary analysis suggests that changes in the criminal-legal system during the COVID-19 pandemic had a detrimental impact on the lives of justice-involved women who use drugs. Participants reported limited access to criminal-legal and social service resources during the COVID-19 pandemic, including community supervision. As a result, women experienced homelessness, hunger, and mental health challenges which fueled increases in substance use. Women’s previous experiences of grief and loss were activated by COVID-19–related death and dying. In addition to these preliminary findings, there were isolated stories that invite further exploration. For example, narratives from women who sold sex during the COVID-19 pandemic described the complications of simultaneously negotiating COVID-19 and HIV safety. Women who were arrested for larceny and released with a “promise to appear” at an unknown future date, used what they perceived to be the inevitability to their future incarceration, and lack of any viable alternatives, to justify further law-breaking and substance use.

Future interviews may offer additional data on the evolving definitions of risk in the context of sex work. Some women reported renting rooms from strangers whom they met in the community, circumstances that could lead to disputes, violence, sexual assault, and arrest. The nuances of this unregulated rental market may be another area of potential discovery in this project. The digital storytelling process, which has not yet begun, will also offer new perspectives and insights into women’s lived experiences.

### Strengths and Limitations

This protocol has several strengths and limitations. First, because all the data will be collected from people living in one northeastern state in the United States, the findings may not be transferable to other communities. Responses to COVID-19 by criminal-legal and social service systems varied across the country. Still, while what happened in this one state may be different than what happened elsewhere, the study will be helpful to researchers and community-based service providers in other regions by suggesting areas of vulnerability that require attention and theorizing about the meanings women ascribe to social policy and administrative actions. Second, the depth and breadth of the findings may be inhibited by the fact that the research team is comprised primarily of undergraduate students with little to no research experience. The pace of the program is shaped by its dual aims: (1) to train new scholars and (2) to build knowledge about complex social phenomenon. At the same time, the team’s composition is a strength. This team of first-time researchers brings novel ideas and enthusiasm that disrupt entrenched practices and knowledge that can inhibit the production of new postulations. Third, the retrospective design of this inquiry raises questions about the accuracy of participants’ memories. Especially if people were engaging in heavy substance use, it can be difficult to recall what transpired during the first year of the COVID-19 pandemic. Given this time lapse, the qualitative design of the study is a strength. This postpositivist methodology recognizes that there are many truths and values storytelling for its ability to produce meaning. In this sense, the most pressing question is, “Why is this story being told?” not “Is this story an accurate description of past events?”

### Dissemination and Future Directions

Dissemination of study findings will take many forms. First, topic summaries will invite conversation with social workers, criminal-legal professionals, and other service providers about what happened during the COVID-19 pandemic and how the community might respond differently to future crises. Two, reflective analysis offers insights into the lives of women who use drugs and narratives that they tell themselves and others about who they are. This nuanced understanding of women’s meaning making and identity that can inform clinical practice and program development. Three, findings will contribute to academic discussions in journals and conferences about engaging undergraduates in research, criminal-legal systems, substance use, and HIV prevention. The focus on women who use drugs and women’s HIV risk in all these dissemination efforts contributes to ongoing efforts to center women’s lives in dialogue about criminal-legal systems, substance use and HIV prevention that tends to focus on men.

Taken together, the multiple methods in this project will build understanding about the lives of women who use drugs during the first year of the COVID-19 pandemic. Findings may inform efforts to reduce reliance on carceral systems by building more community-based programs that to protect and support for vulnerable women.

## Appendix

Multimedia Appendix 1 Report page documenting grant funding.

## Abbreviations

CI: Correctional Institution
NIH/NIDA: National Institutes of Health’s National Institute on Drug Abuse
PI: principal investigator
RA: research assistant
RQI: Rapid Qualitative Inquiry

## Data Availability

The datasets generated and analyzed during this study are not publicly available for reasons of participant confidentiality but are available from the corresponding author on reasonable request.

## References

[1] Fast facts: HIV and women.

[2] HIV and women: HIV diagnosis. Division of HIV Prevention, National Center for HIV, Viral Hepatitis, STD, and TB prevention.

[3] Beckett MK, Collins RL, Burnam MA, Kanouse DE, Bing EG, Longshore DL, Fleishman J, Sherbourne CD, London AS, Turner BJ, Eggan F, Vitiello B, Morton SC, Edelen MO, Bozzette SA, Ortiz-Barron L, Shapiro MF, Bogart LM, Cunningham W, Stein M (2007). Mental health and substance abuse issues among people with HIV: lessons from HCSUS.

[4] Substance Abuse and Mental Health Services Administration [SAMHSA] (2020). Key substance use and mental health indicators in the United States: results from the 2019 National survey on drug use and health (HHS Pub. No. PEP20-07-01-001, NSDUH Series H-55).

[5] Burke JG, Thieman LK, Gielen AC, O'Campo P, McDonnell KA (2005). Intimate partner violence, substance use, and HIV among low-income women: taking a closer look. Violence Against Women.

[6] Meyer JP, Springer SA, Altice FL (2011). Substance abuse, violence, and HIV in women: a literature review of the syndemic. J Womens Health (Larchmt).

[7] Budd KM (2024). Fact sheet: incarcerated women and girls.

[8] (2020). Criminal justice drugfacts.

[9] Maruschak LM, Bronson J, Alper M (2021). Alcohol and drug use and treatment reported by prisoners: survey of prison inmates, 2016.

[10] Carson AE (2020). Prisoners in 2019.

[11] Harawa N, Adimora A (2008). Incarceration, African Americans and HIV: advancing a research agenda. J Natl Med Assoc.

[12] Staton-Tindall M, Leukefeld C, Palmer J, Oser C, Kaplan A, Krietemeyer J, Saum CA, Surratt H (2007). Relationships and HIV risk among incarcerated women. Prison J.

[13] Strathdee SA, West BS, Reed E, Moazen B, Azim T, Dolan K (2015). Substance use and HIV among female sex workers and female prisoners: risk environments and implications for prevention, treatment, and policies. J Acquir Immune Defic Syndr.

[14] Fogel CI, Gelaude DJ, Carry M, Herbst JH, Parker S, Scheyette A, Neevel A (2014). Context of risk for HIV and sexually transmitted infections among incarcerated women in the south: individual, interpersonal, and societal factors. Women Health.

[15] Knittel AK, Shook-Sa BE, Rudolph J, Edmonds A, Ramirez C, Cohen M, Adedimeji A, Taylor T, Michel KG, Milam J, Cohen J, Donohue J, Foster A, Fischl M, Konkle-Parker D, Adimora AA (2020). Incarceration and number of sexual partners after incarceration among vulnerable US Women, 2007-2017. Am J Public Health.

[16] Maru DS, Basu S, Altice FL (2007). HIV control efforts should directly address incarceration. Lancet Infect Dis.

[17] Rowell-Cunsolo TL, Szeto B, Sampong SA, Larson EL (2016). Predictors of sexual behaviour among men and women in New York City area prisons. Cult Health Sex.

[18] Keene DE, Rosenberg A, Schlesinger P, Guo M, Blankenship KM (2018). Navigating limited and uncertain access to subsidized housing after prison. Hous Policy Debate.

[19] Andersen TS (2017). Social support and one-year outcomes for women participating in prison-based substance abuse treatment programming. Crim Justice J.

[20] Knudsen HK, Staton-Tindall M, Oser CB, Havens JR, Leukefeld CG (2014). Reducing risky relationships: a multisite randomized trial of a prison-based intervention for reducing HIV sexual risk behaviors among women with a history of drug use. AIDS Care.

[21] O'Brien P, Young Ds (2006). Challenges for formerly incarcerated women: a holistic approach to assessment. Families in Society: The Journal of Contemporary Social Services.

[22] Morse DS, Wilson JL, McMahon JM, Dozier AM, Quiroz A, Cerulli C (2017). Does a primary health clinic for formerly incarcerated women increase linkage to care?. Womens Health Issues.

[23] Pinto RM, Rahman R, Williams A (2014). Policy advocacy and leadership training for formerly incarcerated women: an empowerment evaluation of ReConnect, a program of the women in prison project, correctional association of New York. Eval Program Plann.

[24] Sawyer W (2019). Who’s helping the 1.9 million women released from prisons and jails each year?.

[25] Bradley R, Davino Km (2002). Women's perceptions of the prison environment: when prison is “The Safest Place I've Ever Been”. Psychol Women Q.

[26] Pattinson T (2015). Prison as a place of safety for women with complex mental health needs.

[27] Burton S, Lynn C (2019). Becoming Ms. Burton: From prison to recovery to leading the fight for incarcerated women.

[28] Kaeble D (2023). Probation and parole in the United States, 2021.

[29] Lorvick J, Comfort Ml, Krebs CP, Kral AH (2015). Health service use and social vulnerability in a community-based sample of women on probation and parole, 2011–2013. Health Justice.

[30] Kang-Brown J, Montagnet C, Heiss J (2021). People in jail and prison in spring 2021.

[31] Widra E How much have COVID-19 releases changed prison and jail populations? Prison Policy Initiative.

[32] Sharma D, Li W, Lavoie D, Lauer C Prison populations drop by 100,000 during pandemic.

[33] (2024). Statistics and monthly indicators.

[34] Glick SN, Prohaska SM, LaKosky PA, Juarez AM, Corcorran MA, Des Jarlais DC (2020). The impact of COVID-19 on syringe services programs in the United States. AIDS Behav.

[35] Perri M, Dosani N, Hwang SW (2020). COVID-19 and people experiencing homelessness: challenges and mitigation strategies. CMAJ.

[36] Benavides AD, Nukpezah JA (2020). How local governments are caring for the homeless during the COVID-19 pandemic. Am Rev Public Adm.

[37] Pilla D, Park-Taylor J (2022). "Halfway Independent": experiences of formerly homeless adults living in permanent supportive housing. J Community Psychol.

[38] Galarneau LR, Hilburt J, O'Neill ZR, Buxton JA, Scheuermeyer FX, Dong K, Kaczorowski J, Orkin AM, Barbic SP, Bath M, Moe J, Miles I, Tobin D, Grier S, Garrod E, Kestler A (2021). Experiences of people with opioid use disorder during the COVID-19 pandemic: a qualitative study. PLoS One.

[39] Fatal unintentional and undetermined intent drug overdose monthly report.

[40] Dunlop A, Lokuge B, Masters D, Sequeira M, Saul P, Dunlop G, Ryan J, Hall M, Ezard N, Haber P, Lintzeris N, Maher L (2020). Challenges in maintaining treatment services for people who use drugs during the COVID-19 pandemic. Harm Reduct J.

[41] Gilbert L, Goddard-Eckrich D, Chang M, Hunt T, Wu E, Johnson K, Richards S, Goodwin S, Tibbetts R, Metsch LR, El-Bassel N (2021). Effectiveness of a culturally tailored HIV and sexually transmitted infection prevention intervention for black women in community supervision programs: a randomized clinical trial. JAMA Netw Open.

[42] Research enhancement awards (R15).

[43] Guest G, Bunce A, Johnson L (2006). How many interviews are enough?: an experiment with data saturation and variability. Field Methods.

[44] Hennink M, Kaiser BN (2022). Sample sizes for saturation in qualitative research: a systematic review of empirical tests. Soc Sci Med.

[45] Beebe J (2014). Rapid qualitative inquiry: A field guide to team-based assessment (2nd ed.).

[46] Goodson I, Antikainen A, Sikes P, Andrews M (2016). The Routledge international handbook on narrative and life history.

[47] Doherty EF (2021). “It’s just like a break-up”: a qualitative analysis of turning points in female friendships in early to middle adulthood. Communication Quarterly.

[48] Brun C, Rapp RC (2001). Strengths-based case management: individuals' perspectives on strengths and the case manager relationship. Soc Work.

[49] Hamby S, McLean KC (2021). Strengths-based approaches to conducting research with low income and other marginalized populations. Cultural methods in psychology: Describing and transforming cultures.

[50] Glasner T, van der Vaart W (2009). Applications of calendar instruments in social surveys: a review. Qual Quant.

[51] Mills K, Emery J, Cheung C, Hall N, Birt L, Walter FM (2014). A qualitative exploration of the use of calendar landmarking instruments in cancer symptom research. BMC Fam Pract.

[52] Lambert J Digital storytelling cookbook.

[53] Digital storytelling online certificate program.

[54] Morra S 8 steps to great digital storytelling.

[55] Birt L, Scott S, Cavers D, Campbell C, Walter F (2016). Member checking: a tool to enhance trustworthiness or merely a nod to validation?. Qual Health Res.

[56] Beebe J (2014). Rapid qualitative inquiry: A field guide to team-based assessment (2nd ed.).

[57] Beebe J (2014). Rapid qualitative inquiry: a field guide to team-based assessment (2nd ed).

[58] Beebe J (2014). Rapid qualitative inquiry: A field guide to team-based assessment (2nd ed.).

[59] Beebe J (2014). Rapid qualitative inquiry: A field guide to team-based assessment (2nd ed.).

[60] Beebe J (2014). Rapid qualitative inquiry: A field guide to team-based assessment (2nd ed.).

[61] Braun V, Clarke V (2023). Toward good practice in thematic analysis: avoiding common problems and be(com)ing a researcher. Int J Transgend Health.

[62] Salmona M, Lieber E, Kaczynski D (2020). Qualitative and mixed methods data analysis using Dedoose: A practical approach for research across the social sciences.

[63] Braun V, Clarke V (2006). Using thematic analysis in psychology. Qual Res Psychol.

[64] Braun V, Clarke V (2023). Toward good practice in thematic analysis: avoiding common problems and be(com)ing a researcher. Int J Transgend Health.

[65] Braun V, Clarke V (2023). Toward good practice in thematic analysis: avoiding common problems and be(com)ing a researcher. Int J Transgend Health.

[66] Braun V, Clarke V (2023). Toward good practice in thematic analysis: avoiding common problems and be(com)ing a researcher. Int J Transgend Health.

[67] Roller MR, Lavrakas PJ (2015). Applied qualitative research design: a total quality framework approach.

[68] Patton MQ (2001). Qualitative research and evaluation methods. (3rd Ed.).

