# Supplementary material for: Impact of Changes in Criminal-Legal Practices During the COVID-19 Pandemic on the HIV Risk Behaviors of Women Who Use Drugs: Protocol for a Multimethods Qualitative Study
Source: JMIR Res Protoc. 2024 Dec 20;13:e58285. doi: 10.2196/58285 (PMC11699493; doi:10.2196/58285)
Supplement: Multimedia Appendix 1 [file resprot_v13i1e58285_app1.pdf]

NIH RePORTER Announcement:

Cooperative Agreement Search now available on RePORTER! A new Cooperative Agreement field is displayed on the Advanced Search page. In addition, Cooperative Agreement is also a selection in the Activity Code dropdown.

Project Details

Share

Description
Details
Sub-Projects
Publications
Patents
Outcomes
Clinical Studies
News and More
History
Similar Projects

Impact of COVID-19 Criminal-Legal Practices on HIV Risk among Women Who Use Drugs: A Multi-Methods Qualitative Exploration

Project Number	Contact PI/Project Leader	Awardee Organization
1R15DA056285-01	SMOYER, AMY BROOKS	SOUTHERN CONNECTICUT STATE UNIVERSITY

Description

Abstract Text

Project Summary/Abstract The goal of this R15 Academic Research Enhancement Award (AREA) study is to build knowledge about the impact of COVID-19 shifts in criminal-legal practices on the HIV risk behaviors of justice-involved women who use drugs (WWUD). WWUD are disproportionately impacted by HIV. Incarceration has been identified as one of the myriad individual and structural factors that elevates the HIV risk of WWUD. During the COVID-19 pandemic, the number of women incarcerated in Connecticut was drastically reduced to avoid prison crowding. This change in criminal-legal practices created an opportunity to build knowledge about the impact of non-incarceration responses to illicit behavior on the HIV risk of WWUD. The specific aims of the study are to (1) Provide undergraduate students at Southern Connecticut State University the opportunity to gain significant experience with community engaged research; (2) Identify the pre-COVID-19 HIV risk behaviors and protective factors of justice-involved WWUD; (3) Document and assess HIV risk during COVID-19 among justice-involved WWUD. Multiple methods will be used in this longitudinal qualitative study. First, qualitative life history interviews will be conducted with 30 formerly incarcerated adult WWUD in New Haven, CT, in order to understand and describe their lived experiences and HIV risk prior to COVID-19. This data will create a baseline understanding about their psychosocial conditions and interactions with criminal-legal systems. Next, the same sample of women will participate in individual calendar-based interviews in order to document their HIV risk and interactions with criminal-legal systems during COVID-19 (March 2020 – March 2022). After these interviews have been conducted, a sub-sample of 10 study participants will participate in an 8-week digital storytelling program. The digital stories produced during this stage of the study will offer additional data about participants' lives during COVID-19 that will triangulate the calendar data and offer a creative tool for sharing findings with community. Rapid Qualitative Inquiry methods will be used to collect, manage, and analyze data in ways that centers team-based work, insiders' perspectives, and iterative practices that facilitate rapid understanding and dissemination of findings. All aspects of this study will be developed and conducted by a diverse team of undergraduates in partnership with community consultants who have been personally impacted by incarceration and drug use. Students will meet regularly with the PI and consultants, conduct 12 interviews each, co-facilitate the digital storytelling project, analyze data, author papers, and disseminate findings. A website will be developed to share preliminary findings and final analyses, and the study team will present findings at academic conferences, community forums, and through peer-reviewed journals. By diversifying the research workforce and analyzing the COVID-era experiences of WWUD, this project aligns with NIH's commitment to social science research that addresses the structural determinants of HIV risk.

Was this page helpful? Yes No

Public Health Relevance Statement

Project Narrative Our study will examine the impact of COVID-19 shifts in criminal-legal practices on the HIV risk behaviors of justice-involved women who use drugs. This project brings attention to the structural determinants of HIV risk by exploring how not incarcerating women who use drugs shapes their HIV risk. This knowledge about the lived experiences of women who use drugs during COVID-19 will inform community-based HIV prevention interventions.

NIH Spending Category

Basic Behavioral and Social Science
Behavioral and Social Science
Clinical Research
Coronaviruses
Coronaviruses Disparities and At-Risk Populations
Drug Abuse (NIDA only)
Emerging Infectious Diseases
HIV/AIDS
Health Disparities
Infectious Diseases
Prevention
Social Determinants of Health
Substance Misuse
Women's Health

Project Terms

AIDS prevention
Academic Research Enhancement Awards
Acute
Address
Adult
Attention
Behavior
COVID-19
COVID-19 impact
COVID-19 pandemic
COVID-19 prevention
Calendar
Communities
Connecticut
Consensus
Correctional Institutions
Crowding
Data
Drops
Drug usage
Economics
Employment
Environment
Goals
HIV
HIV risk
Harm Reduction
Healthcare
Housing
Imprisonment
Incidence
Individual
Interpersonal Relations
Interruption
Interview
Journals
Justice
Knowledge
Lead
Left
Legal
Legal system
Mediating

Read More

Details

Contact PI/ Project Leader

Name
SMOYER, AMY BROOKS

Title
ASSISTANT PROFESSOR

Contact

View Email

Other PIs

Not Applicable

Program Official

Name
LEE-WINN, ANGELA EUNJI

Contact

View Email

Organization

Name
SOUTHERN CONNECTICUT STATE UNIVERSITY

City
NEW HAVEN

Country
UNITED STATES (US)

Department Type
NONE

Organization Type
UNIVERSITY-WIDE

State Code
CT

Congressional District
03

Other Information

Opportunity Number
PAR-21-155

Study Section
HIV/AIDS Intra- and Inter-personal Determinants and Behavioral Interventions Study Section[HIBI]

Fiscal Year
2022

Award Notice Date
18-April-2022

Administering Institutes or Centers
National Institute on Drug Abuse

CFDA Code
279

DUNS Number
145900825

UEI
HJL7S6MUNL85

Project Start Date
01-May-2022

Project End Date
30-April-2025

Budget Start Date
01-May-2022

Budget End Date
30-April-2025

Project Funding Information for 2022

Total Funding
$414,643

Direct Costs
$300,000

Indirect Costs
$114,643

Year	Funding IC	FY Tota
2022	National Institute on Drug Abuse	$414,643

NIH Categorical Spending

Click here for more information on NIH Categorical Spending

Funding IC	FY Total Cost by IC	NIH Spending Category
NATIONAL INSTITUTE ON DRUG ABUSE	$414,643	Basic Behavioral and Social Science; Behavioral and Social Science; Clinical Research; Coronaviruses; Coronaviruses Disparities and At-Risk Populations; Drug Abuse (NIDA only); Emerging Infectious Diseases; HIV/AIDS; Health Disparities; Infectious Diseases; Prevention; Social Determinants of Health; Substance Misuse; Women's Health

Sub Projects

No Sub Projects information available for 1R15DA056285-01

Publications

› Disclaimer

No Publications available for 1R15DA056285-01

Patents

No Patents information available for 1R15DA056285-01

Outcomes

The Project Outcomes shown here are displayed verbatim as submitted by the Principal Investigator (PI) for this award. Any opinions, findings, and conclusions or recommendations expressed are those of the PI and do not necessarily reflect the views of the National Institutes of Health. NIH has not endorsed the content below.

No Outcomes available for 1R15DA056285-01

Clinical Studies

No Clinical Studies information available for 1R15DA056285-01

News and More

Related News Releases

No news release information available for 1R15DA056285-01

History

Export

Total project funding amount for 1 project is $414,643*

** Only NIH, CDC and FDA funding data*

Project Number	Sub	Principal Investigator(s)/ Project Leader(s)	Organization				Fiscal Year
**Impact of COVID-19 Criminal-Legal Practices on HIV Risk among Women Who Use Drugs: A Multi-Methods Qualitative Exploration**							
1R15DA056285-01		SMOYER, AMY BROOKS	SOUTHERN CONNECTICUT STATE UNIVERSITY	2022	NIDA	NIDA	$414,643

Similar Projects

Match Score	Project Number	Sub	Principal Investigator(s)/ Project Leader(s)	Organization			
**Minimal Standards of Adequacy: A History of Health Care in US Prisons**							
325	5G13LM013552-03		ADLER, JESSICA L.	FLORIDA INTERNATIONAL UNIVERSITY	2024	NLM	NLM
**Mechanisms underlying recurrent bacterial vaginosis and HIV risk A Multidisciplinary app**							
364	5R01AI138718-05		ALCAIDE, MARIA LUISA	UNIVERSITY OF MIAMI SCHOOL OF MEDICINE	2023	NIAID	NIAID
**The Impact of COVID-19 on End-of-Life Care for Vulnerable Populations**							
411	5R01NR019792-03		ALDRIDGE, MELISSA DIANE	ICAHN SCHOOL OF MEDICINE AT MOUNT SINAI	2023	NINR	NINR
**Integration of Electronic SBI(RT) into an HIV Testing Program to Reduce Substance Use ar Behavior among MSM in Argentina**							
430	5R34DA055503-02		BALAN, IVAN C	FLORIDA STATE UNIVERSITY	2023	NIDA	NIDA
**A Preconception Health Intervention to Reduce Substance Exposed Pregnancies among Ir Women**							
353	5K23DA053433-03		BELLO KOTTENSTETTE, JENNIFER	SAINT LOUIS UNIVERSITY	2024	NIDA	NIDA
**Effects of methamphetamine use on risk behavior, systemic and mucosal inflammation, ar transmitted infection (STI)/HIV risk among men who have sex with men**							
355	5K23DA054004-03		BLAIR, CHERIE SAVINE	UNIVERSITY OF CALIFORNIA LOS ANGELES	2023	NIDA	NIDA
**Early education and later achievement: The impact of COVID-19 on educational attainmen American children**							
351	5R01MD016085-02		BLANKSON, ARABA NAYENA	SPELMAN COLLEGE	2023	NIMHD	NIMHI
**Serological and functional impact of COVID-19 vaccination on the maternal fetal unit and i immunity**							
413	5R21HD107761-02		BOELIG, RUPSA CHAUDHURY [illegible]	THOMAS JEFFERSON [illegible]	2023	NICHD	NICHD
